# A Qualitative Study of Barriers to Enrollment into Free HIV Care: Perspectives of Never-in-Care HIV-Positive Patients and Providers in Rakai, Uganda

**DOI:** 10.1155/2013/470245

**Published:** 2013-08-22

**Authors:** Gertrude Nakigozi, Lynn Atuyambe, Moses Kamya, Fredrick E. Makumbi, Larry W. Chang, Neema Nakyanjo, Godfrey Kigozi, Fred Nalugoda, Valerian Kiggundu, David Serwadda, Maria Wawer, Ronald Gray

**Affiliations:** ^1^Rakai Health Sciences Program, P.O. Box 279, Kalisizo, Rakai, Uganda; ^2^Department of Community Health and Behavioral Sciences, Makerere University School of Public Health, P.O. Box 7072, Kampala, Uganda; ^3^Department of Medicine, Makerere University College of Health Sciences, P.O. Box 7072, Kampala, Uganda; ^4^Department of Epidemiology and Biostatistics, Makerere University School of Public Health, P.O. Box 7072, Kampala, Uganda; ^5^Johns Hopkins School of Medicine, 600 N. Wolfe Street, Baltimore, MD 21287, USA; ^6^Department of Disease Control and Environmental Health, Makerere University School of Public Health, P.O. Box 7072, Kampala, Uganda; ^7^Department of Epidemiology, Johns Hopkins Bloomberg School of Public Health, 627 N. Washington Street, Baltimore, MD 21205, USA

## Abstract

*Background*. Early entry into HIV care is low in Sub-Saharan Africa. In Rakai, about a third (31.5%) of HIV-positive clients who knew their serostatus did not enroll into free care services. This qualitative study explored barriers to entry into care from HIV-positive clients who had never enrolled in care and HIV care providers. *Methods*. We conducted 48 in-depth interviews among HIV-infected individuals aged 15–49 years, who had not entered care within six months of result receipt and referral for free care. Key-informant interviews were conducted with 12 providers. Interviews were audio-recorded and transcripts subjected to thematic content analysis based on the health belief model. *Results*. Barriers to using HIV care included fear of stigma and HIV disclosure, women's lack of support from male partners, demanding work schedules, and high transport costs. Programmatic barriers included fear of antiretroviral drug side effects, long waiting and travel times, and inadequate staff respect for patients. Denial of HIV status, belief in spiritual healing, and absence of AIDS symptoms were also barriers. *Conclusion*. Targeted interventions to combat stigma, strengthen couple counseling and health education programs, address gender inequalities, and implement patient-friendly and flexible clinic service hours are needed to address barriers to HIV care.

## 1. Introduction

There is a need for all HIV-positive persons to access care and preventive interventions in a timely manner [1–4] and to prevent HIV transmission to partners and morbidity and mortality [5–7]. However, uptake of free HIV care services after diagnosis remains suboptimal. A study in Uganda showed that 31.5% of HIV-positive persons who knew their HIV status had not enrolled into care six months after testing HIV positive [8]. Similar findings have been observed in the United States [9–12]. Studies in Sub-Saharan Africa (SSA) to identify barriers to entry into HIV care have focused on those already enrolled into care [13–16]. However, it is critical to directly ascertain information on such barriers from HIV-positive people who are not under care, because their views could be more relevant to the design of targeted interventions to improve HIV care utilization. In order to understand barriers to entry into HIV care by the HIV-positive persons not in care, we conducted a qualitative evaluation among HIV-positive people and health care providers in Rakai, Uganda.

## 2. Materials and Methods

### 2.1. Study Design

This was a qualitative study nested within a study of nonenrollment into free community HIV care in Rakai, Uganda [8].

### 2.2. Study Setting and Participants

Study participants were drawn from the Rakai Community Cohort Study (RCCS), an annual surveillance of consenting/assenting persons aged 15 to 49 years in 50 villages in rural Rakai district, South Western Uganda. The RCCS has close to 1400 HIV-infected participants, the majority of whom (over 90%) know their HIV status and have received counseling and referral [8]. All RCCS participants who provide blood for HIV testing have free access to HIV counseling either as individuals or couples by trained Rakai program counselors in the participant's home or at community-based counseling facilities. HIV-positive individuals who accept their HIV results receive posttest counseling and education on the need to enroll for HIV care and are referred to the Rakai Program HIV/AIDS (Suubi or “hope”) clinic closest to their residence. HIV-positive participants for this study were selected from datasets of persons who had received their HIV-positive results but had not yet enrolled into care six months after receipt of their HIV results and referral [8]. These individuals were actively traced through the HIV counselors, using the address they provided at the most recent RCCS interview.

### 2.3. Data Collection and Sampling Strategy

Between September 2010 and January 2011, we conducted 48 in-depth interviews (IDIs) among consenting/assenting clients from 15 to 49 years, who had received their HIV diagnosis but had not enrolled for HIV care within six months after referral for free HIV services. 12 key-informant interviews (KIIs) were conducted with health care providers (3 HIV counselors, 3 home visitor nurses, and 6 peer educators). Participants for both the IDIs and KIIs were purposively selected based on their availability at time of tracing and willingness/ability to provide written informed consent/assent prior to interview. Informed consent/assent and permission to record the interview were obtained from all participants. Interviews were conducted by trained research assistants with experience in qualitative data collection. Interviews were conducted in Luganda (the most commonly spoken local language in Rakai). IDIs lasted approximately one hour and KIIs about 40 minutes. All interviews were audio-recorded and transcribed. We used in-depth interviews instead of focus group discussions so as to obtain detailed contextualized information as well as to avoid inadvertent HIV status disclosure.

### 2.4. Data Management and Analysis

All interviews were transcribed then translated into English by the interviewer. A 10% back translation was done for quality control. Transcript review began while data collection was still underway, and participant responses were used to shape future interview questions. Verbatim transcripts and sociodemographic information were used for analysis. Two independent coders used the word processed text to develop a codebook by categorizing responses from the interviews to identify themes generated from the in-depth and KIIs about barriers to enrollment into HIV care. Themes were not predetermined but emerged through data review. The coded passages were collapsed into categories and grouped in accordance with the health belief model (HBM) to conceptualize determinants of failure to enroll into HIV care services. These HBM concepts included perceived susceptibility to HIV, perceived severity of HIV disease and perceived barriers to enrollment for care. Transcripts were subjected to thematic content analysis using Atlas-ti version 5.5.9. Quotations from the HIV-positive persons are denoted by type of interview (IDI), sex, and age, while those for providers are denoted by KII and type of provider.

### 2.5. The Health Belief Model (HBM)

The health belief model (HBM) is a psychological model that attempts to explain and predict health behaviors, by focusing on the attitudes and beliefs of individuals. The basic structure of the model is derived from psychological theories suggesting that health-seeking behaviors are determined at the individual level by a desire to avoid illness and a belief that a particular course of action will prevent or relieve illness [17]. The HBM uses constructs representing the perceived threat and net benefits such as perceived *susceptibility*, *severity*, *benefits*, *barriers*, *cues to action* (*the concept of* “readiness to act”), and *self-efficacy* or confidence in the ability to successfully perform an action. The model has previously been used to describe clinic attendance, as well as HIV-related behaviors among various populations including those in African settings [18]. In this study, the HBM was applied to conceptualize the individual and social determinants of enrollment into care among un-enrolled HIV-infected individuals. 

### 2.6. Ethical Considerations

Approval for the study was obtained from the Makerere University School of Public Health Higher Degrees Research and Ethics Committee and the Uganda National Council of Science and Technology. All study participants were informed of study procedures, risks, and benefits and provided written consent/assent for enrollment. Research assistants were well trained in the conduct of qualitative data collection and the ethical issues in human research. Participants received unique study numbers and data sets contained no personal identifiers. Data was stored in password-protected computers accessible only by authorized personnel. IDI and KII participants were assured of confidentiality, and each interview was conducted in private.

## 3. Results

### 3.1. Informant Characteristics

A total of 48 HIV-positive participants who had not enrolled into HIV care six months or more following HIV diagnosis were contacted and completed qualitative interviews. Of the 48 participants, 62.5% were 26 years or older, 52% were male, 64.6% were married, and 48% had postprimary education. There were 12 HIV care providers (three HIV counselors, three home visitor nurses, and six peer educators).

### 3.2. Perceived Barriers to Utilizing HIV Care Services

Stigma, defined as a social process, experienced or anticipated, characterized by exclusion, rejection, blame, or devaluation that results from experience, perception, or anticipation of an adverse social judgment about a person or group [19] emerged as a major determinant of nonenrollment into HIV services among nineteen of the participants (39.6%). Stigma was manifested as expressions of fear of being seen by other people at the HIV clinic, hence inadvertently disclosing their HIV status. Participants anticipated disrespect, ill treatment, or social isolation by their community or peers, if known to be HIV infected. Entry into HIV care was associated with fear of exclusion from participation in community social and political events or falling out with a social or peer group. One participant recounted his apprehension about entry into care.

**I feared. I thought that if I go for care and others see me they will begin to despise me, they will stop giving me respect. (IDI, Male, 23)**

Among ten participants (20.8%), disclosure of one's positive HIV status and entry into HIV care was linked to negative consequences such as loss of a sexual partner or marital dissolution and family rejection. Participants also reported the fear of losing social support from caretakers if they disclosed their HIV status by entering HIV care. 

Some providers highlighted that lack of support from the male sexual partner negatively influenced the woman's utilization of HIV care services. Two providers (16.7%) reported the challenges faced by female clients, especially those in HIV-discordant relationships, when making decisions to utilize HIV care services. Male partners prevented their spouses from going to HIV clinics in order to conceal the man's HIV status or to prevent perceived wastage of household resources. Women feared that acting contrary to the male partner's directive would result in domestic violence or marital dissolution.

**Others are refused to come to the clinic by their partners. He may refuse the wife to come to the “Suubi” clinic. He may choose to chase away the wife if she insists on enrolling for HIV care, so the wife may fear separation. (KII, HIV counselor)**

Despite being aware of the availability of HIV care services, two (4%) female participants did not seek care due to competing family obligations including looking after sick family members, obtaining food, and caring for young children and domestic animals. Such roles in a household were primarily performed by women and, in the absence of another female to take over the chores, leaving home to seek HIV care was perceived as impractical.

Absence from home in search of work was a hindrance to entry into care reported by five (10%) of the male participants, whose main occupation was fishing or trading. Their work involved frequent travel hence increasing the distance to the HIV clinics and limiting the time available for clinic attendance. This, coupled with the disparity between the work and clinic schedules, made it extremely difficult to utilize HIV services.

**Most people get overwhelmed by work. When they find themselves working far away from their homes they fail to attend clinics. The nature of work I do keeps me so busy and I move away from home and go for example, to Kampala. When I get there I may take long without returning home. I really travel a lot. (IDI, Male, 31)**

For some of the participants, the long distance, coupled with lack of transport posed a major barrier to entry into HIV care. Eight study participants (16.7%) cited long distance to the clinic and high transport costs as the main barriers to using HIV care. Without personal transport, they would have to rely on friends or public transportation, which had irregular schedules and were expensive. Three providers (25%) agreed that transportation costs were a major hindrance to HIV care utilization.

**What brings problems is the issue of the transport. Some people may fail to raise transport to the clinics and in a way fail to enroll for HIV care. (IDI, Male, 22)**

**Some clients have failed because of the distance. When they imagine walking long distances and yet he or she is feeling unwell they decide not to come. The problem is long distances. (KII, HIV counselor)**

The fear of life-long HIV medication and its side effects prevented eight individuals (16.7%) from entering care. The need for strict drug dosing schedules created a fear of failure to adhere to health worker recommendations. The perceptions about HIV medications were largely based on reported experiences of other HIV-infected individuals who had received HIV medications.

**Some people fear that once you begin on medication it can accelerate death. However, it may be a wrong perception but I am not sure. Some people also complain that some drugs are hard to take. This scares some people away from HIV health care. (IDI, Female, 23)**

Two participants and one provider reported that the long waiting hours at the clinic were a barrier to entry into care. Patients were required to come to the clinic early, but it was not unusual for a patient to leave several hours later, especially if his/her clinic visit involved many activities. One participant narrated how the length of time spent at the clinic by some of his HIV-positive colleagues had dissuaded him from entering HIV care.

**Patients take the whole day there [at the clinic] one may go there at 8 : 00 am and come back at 4:00 pm. This has not encouraged me to go to the clinic. (IDI, Male, 24)**

**Most of our clients complain about the time they spend at the clinic. They say that you have to sacrifice the whole day and forego a lot of work. The time they spend here is much and this delays them to enroll. (KII, HIV counselor)**

Anticipated disrespect from some care providers prevented two participants (4.2%) from using HIV care services. These informants reported that some patients felt devalued by providers, especially through reprimands.

### 3.3. Perceived Susceptibility to HIV Disease

A person's assessment of the likelihood of succumbing to HIV-related infections or death led to denial of HIV status. One study participant and one provider reported that doubts about the accuracy of HIV status were a barrier to enrollment for HIV care. One HIV-positive person had received HIV testing and been counseled but doubted his status because he was still in good health and physically strong, as reported below.

**I feel strong and sometimes doubt the positive HIV test results. I also have a friend who told me yesterday that he has never had any sign or feeling of being unhealthy. He has been strong all the time but the health workers say he has HIV. I feel reluctant after I doubt my HIV status. (IDI, Male, 25)**

Belief in spiritual healing by God was another barrier to entry into medical care. One male Pentecostal believer assumed he had been healed by God and even reported better physical health than before.

**Before I had an HIV test I used to suffer from a lot of opportunistic infections like body itches and, persistent fever. I decided to take an HIV test and they found that I had HIV. From that time I read the scriptures and felt strong that God can heal me in the name of Jesus Christ. I begun prayer and got healed of all the infections without use of any drugs. (IDI, Male, 44)**

### 3.4. Perceived Severity of HIV

This concept is based on the HIV-positive person's judgment of how serious HIV infection and its consequences are to his/her health. Feeling healthy was raised as a major reason for not seeking HIV medical care. Seven study participants (14.6%) felt that seeking medical care only became necessary when one felt sick or had symptoms and signs of HIV disease. The absence of physical symptoms diminished the need to seek care.

**I did not feel sick. That is the main reason why I did not enroll. I will enroll at Suubi clinic when I start falling sick or if I see any sign. (IDI, Female, 25)**

## 4. Discussion

This study explored barriers to enrollment for free HIV care in Rakai, Uganda, using the health belief model to assess factors affecting nonentry into care. Enrollment into care was defined as entry of HIV-positive persons into HIV care within six months of receipt of positive HIV test results and referral to HIV care services. The six-month cutoff was selected as a marker of programmatically significant delay in care seeking. A prior study indicated that 34.1% of ART-eligible patients who did not initiate ART died within six months [7].

Perceived barriers to using HIV care services included social concerns like the fear of stigma or the negative consequences of HIV status disclosure, lack of support from the male partner or household head, and competing family responsibilities; economic barriers like work schedules and high transport costs; patient fear of HIV medications and long clinic waiting time, distance to the clinic, and inadequate respect for patients by clinic staff. Perceived susceptibility to HIV led to denial of HIV status and belief in spiritual healing of HIV, while the absence of physical HIV symptoms was perceived to decrease the need for care. There was considerable agreement on factors influencing failure to enter HIV medical care between the patients and providers, unlike another study [20] which found a mismatch between patient and healthcare provider perceptions of barriers.

Stigma as a perceived barrier to using HIV care services is consistent with findings from other studies that implicated HIV-related stigma and discrimination toward people living with HIV (PLWH) as a barrier to HIV care [20–26]. This suggests that three decades into the HIV epidemic, stigma still remains central in the experiences of PLWH. There is a need to fight stigma at individual, family, and community levels. The role of HIV posttest clubs and networks to provide social support and combating stigma should be emphasized.

The fear of disclosure of HIV status and its accompanying risks prevented some patients from enrolling for care. Other studies have also highlighted this fear and stress and the complexities of HIV disclosure to sexual partners [27]. Encouraging couple counseling and testing [28] as well as counselor-assisted HIV status disclosure [29] could help PLWHA disclose their HIV status to their partners.

The role of men in their female partners' decision to use HIV care services is important in this setting, where approximately 70% of the households are headed by men, with the power and control over allocation of resources [30]. Some women were prevented from enrolling into HIV care by their partners. This finding is consistent with another study in Zimbabwe [31]. In Africa, gender inequality remains a barrier for women's access to treatment and other health services [32]. It is therefore important to address gender inequalities and household dynamics that prevent women from entering care. Women's utilization of HIV care services is further limited by their gender roles in the home. Lack of time is a barrier for many women, and domestic responsibilities frequently compete with their own health care needs [33, 34].

Participants also reported dissatisfaction with some of the clinic processes, especially the long waiting times. This was a major deterrent especially among patients involved in the cash economy, because lost time directly translated into lost earnings. These findings suggest a need to institute more flexibility in hours of service to accommodate persons with demanding work schedules.

Although HIV services are provided free of cost, transport costs, as in previous studies [20, 23, 24, 35], were a critical barrier to accessing HIV care. Decentralization of HIV care via outreach HIV services or compensation for transport costs could mitigate this barrier.

Perceptions of callous treatment by health workers were associated with nonenrollment into care as has been reported in other settings [23, 36–38]. Personnel providing HIV care services need to be trained in the importance of empathy towards the patients and courtesy, as well as engaging patients as partners in the HIV care process.

Our results suggest a need to reexamine the quality and content of posttest counseling when communicating positive HIV test results. To improve linkage to care, there is a need to address perceptions of risk with HIV medications and trust in the health care system and in health care workers. The negative beliefs about HIV medications can be addressed through health education during posttest counseling. Ongoing support by HIV counselors or HIV-positive peer educators may facilitate linkage to care for persons with concerns about medications [39].

In line with the health belief model, perception of susceptibility to HIV contributed to decisions not to access care. Denial of HIV status, as identified in earlier studies [23, 35] needs to be addressed through interventions that support acceptance of HIV status and the implications of the diagnosis. Some participants believed in spiritual healing and hence did not seek modern care. Spiritual beliefs impact care for other chronic illnesses and HIV treatment adherence [40–42]. Spiritual beliefs should be an important part of HIV counseling, and there is need to encourage close collaboration between HIV care programs and religious institutions, so as to provide appropriate messages to HIV-infected patients.

Participants avoided HIV care because of absence of physical symptoms. Being asymptomatic makes it difficult for some persons to believe that they are HIV infected [35, 43, 44] and they only seek care after the onset of symptoms [11, 45]. This finding demonstrates how one's perception of illness affects the decision to seek care. Health education and counseling on the importance and benefits of early entry into care should be emphasized.

The use of qualitative methods and the inclusion of both patients' and HIV care providers' views is strength to this study. A potential limitation to this study is that the objective of the research was exploratory, and hence, the findings may not be generalizable. Also, due to logistical constraints, we interviewed only participants who could be located and these may differ from those that were difficult to locate.

## 5. Conclusion

 Our study suggests several key barriers to enrollment into HIV care among persons diagnosed with HIV. To improve entry into HIV care, comprehensive interventions are needed to combat stigma, encourage couple HIV counseling, address gender inequalities, strengthen health education and counseling programs, and implement more patient friendly and flexible clinic service hours. Without targeted interventions to address these barriers, engagement in care for PLWH will likely remain inadequate.
